# Long‐read sequencing reveals genomic and epigenomic variation in the dark genome of human Alzheimer's disease

**DOI:** 10.1002/alz.70852

**Published:** 2025-11-14

**Authors:** Paulino Ramirez, Wenyan Sun, Shiva Kazempour Dehkordi, Habil Zare, Giovanni Pascarella, Piero Carninci, Bernard Fongang, Kevin F. Bieniek, Bess Frost

**Affiliations:** ^1^ Barshop Institute for Longevity and Aging Studies San Antonio Texas USA; ^2^ Glenn Biggs Institute for Alzheimer's and Neurodegenerative Diseases San Antonio Texas USA; ^3^ Department of Cell Systems and Anatomy University of Texas Health San Antonio San Antonio Texas USA; ^4^ Center for Alzheimer's Disease Research Brown University Providence Rhode Island USA; ^5^ Clinical Neuroscience Research Center Department of Neurosurgery School of Medicine Tulane University New Orleans Louisiana USA; ^6^ Laboratory for Transcriptome Technology RIKEN Center for Integrative Medical Sciences Yokohama Kanagawa Japan; ^7^ Department of Biochemistry & Structural Biology University of Texas Health San Antonio San Antonio Texas USA; ^8^ Department of Pathology University of Texas Health San Antonio San Antonio Texas USA

**Keywords:** Alzheimer's disease, epigenomics, genomics, repetitive elements

## Abstract

**INTRODUCTION:**

Faulty DNA repair and epigenetic regulation contribute to neurodegeneration in Alzheimer's disease. Long‐read sequencing enables analysis of “dark regions” that are difficult to study via traditional sequencing methodologies.

**METHODS:**

Using nanopore whole‐genome DNA sequencing of *post mortem* brain from early‐ and late‐ stage Alzheimer's disease cases and controls, we analyzed retrotransposition, non‐allelic homologous recombination (NAHR), structural variation, and DNA methylation within repetitive regions.

**RESULTS:**

Retrotransposon insertions and NAHR were enriched in centromeric/pericentromeric and ribosomal DNA (rDNA) in the aged brain. Putatively somatic AluY retrotransposon insertions trended upward in late‐stage disease. Enrichment of NAHR between repetitive regions and DNA demethylation were detected in early disease. Differential methylation of dark regions within specific Alzheimer's disease risk genes and repetitive elements occurred across disease stage.

**DISCUSSION:**

This study provides the first long‐read analysis of repetitive elements in the aged human brain and identifies these regions as hotspots for genomic variation in Alzheimer's disease.

**Highlights:**

- Long‐read sequencing enables analysis of “dark” regions Alzheimer's disease brains
- Somatic AluY retrotransposon insertions may be elevated in late‐stage disease
- Retrotransposon‐associated non‐allelic homologous recombination (NAHR) is enriched in repetitive regions in early disease
- DNA demethylation within the centro/pericentromere and ribosomal DNA (rDNA) occur across disease stage
- Dark regions of risk genes are differentially methylated across disease stage

## 1 INTRODUCTION

While landmark studies have revealed genetic associations contributing to Alzheimer's disease risk,^1^, ^2^, ^3^ the inability of traditional DNA sequencing methods to analyze genomic “dark regions” limits our understanding of the full genetic architecture of disease. Dark regions are highly repetitive elements that are not resolved via traditional 50–150 bp sequencing,^4^, ^5^, ^6^, ^7^, ^8^ many of which are lacking in the GRCh38 human reference genome. These regions include low‐complexity microsatellites, transposable element‐rich sequences, centromeric DNA, and rDNA arrays. Combining the new human CHM13 telomere‐to‐telomere reference genome with long‐read DNA sequencing overcomes this challenge by generating long reads that enable confident mapping to repetitive loci.^9^ Nanopore sequencing of native, un‐amplified DNA further facilitates analysis of DNA modifications such as 5‐methylcytosine (5mC) within repetitive sequences.

The brain accumulates structural variants that result in genomic mosaicism between cells as organisms age.^10^ Structural variants can occur in progenitor cells, proliferating glial and epithelial cells, and in post‐mitotic neurons, and can arise from errors in DNA repair, homologous recombination, and retrotransposition. Retrotransposition is a process in which retrotransposon‐encoded RNA is reverse transcribed into complementary DNA integrates back into the genome, generating a novel insertion.^11^ Studies in *Drosophila*, cultured cells, mouse brain, and human Alzheimer's disease report that retrotransposon RNA is significantly elevated in settings of tauopathy.^12^, ^13^, ^14^ While evidence in *Drosophila* and HeLa cells suggests that pathogenic forms of tau cause retrotransposition,^13^, ^15^ it is currently unknown if retrotransposons mobilize to a greater degree in the human brain affected by Alzheimer's disease.

While retrotransposons make up 35% of the human genome, most retrotransposons have lost the ability to mobilize due to mutations that have accumulated over the course of evolution. Specific subfamilies of long interspersed nuclear element (LINE) retrotransposons are capable of autonomous retrotransposition in humans; specific families of short interspersed nuclear element (SINE) and SINE‐VNTR‐Alu (SVA) retrotransposons are active and leverage LINE‐encoded protein for trans‐retrotransposition.^16^, ^17^, ^18^, ^19^ Retrotransposition of the active human‐specific LINE‐1 element (L1Hs) occurs in neurons and glia and is a proposed physiological feature of developing neurons.^20^, ^21^, ^22^ In addition to *de novo* retrotransposition events, the repetitive nature of retrotransposon DNA has the potential to facilitate non‐allelic homologous recombination (NAHR), leading to new genomic variants.^23^, ^24^, ^25^, ^26^, ^27^ NAHR, documented in Alzheimer's disease,^28^ can arise from double stranded DNA breaks that occur during instances of high cellular stress and contribute to the loss of genomic integrity.^28^

Here we utilized Oxford Nanopore Technologies native DNA sequencing to identify putatively somatic retrotransposition events, NAHR, structural variants, and DNA methylation changes in frontal cortex of individuals at early and late stages of Alzheimer's disease and age‐matched controls, with a particular focus on dark regions of the human genome. The pathological distribution of tau in Alzheimer's disease is defined by Braak neurofibrillary tau tangle staging. Compared to a brain lacking tau tangles (Braak 0), tau pathology progresses from brainstem to transentorhinal/entorhinal regions (Braak I), to the primary hippocampus (Braak III) and then association and primary neocortical regions of the brain (Braak V‐VI).^29^ Among all brains analyzed, we find that repetitive segments of the genome are particularly susceptible to genomic changes compared to coding regions. Putatively somatic retrotransposon insertions primarily occur within centromeric and pericentromeric regions of the aged brain. While NAHR is also elevated in centromeric and pericentromeric regions, we find that 47S rDNA exhibits a particularly high frequency of NAHR compared to other genomic regions. In cases with Alzheimer's disease, we detect a trending increase in rare, putatively somatic retrotransposition events involving the SINE AluY family in advanced disease, as well as disease stage‐specific differences in NAHR and DNA methylation of repetitive elements and retrotransposons. Overall, our study represents the first long‐read DNA sequencing‐based analysis of retrotransposons, NAHR, structural variants, and DNA methylation in genomic dark regions in brains affected by Alzheimer's disease and identifies retrotransposons, centromeric/pericentromeric regions and rDNA as key hotspots of genomic variation.

## 2 METHODS

### 2.1 Sample collection

18 *post mortem* frontal cortex samples were selected to capture equal numbers of Braak stages 0, III, and V/VI with an average age of 76.6 years (68–92). Samples were Caucasian and were relatively balanced for sex and age with respect to Braak group. Samples were provided by Dr Dennis Dickson of the Mayo Clinic Brain Bank or acquired from the NIH NeuroBioBank.

**RESEARCH IN CONTEXT**

1. **Systematic review**: Evidence suggests that defects in DNA repair and epigenetic control mediate neurodegeneration in Alzheimer's disease. DNA sequencing studies have been applied to study these processes but are unable to characterize dark regions of the genome. Advances in sequencing technologies now enable more comprehensive genome analyses.
2. **Interpretation**: Analysis of retrotransposon insertions and non‐allelic homologous recombination (NAHR) revealed enrichment in centromeric/pericentromeric and ribosomal DNA (rDNA) in the aged brain. Putatively somatic AluY retrotransposon insertions trended upward in late‐stage disease. DNA demethylation in centro/pericentromeres and rDNA, along with differential methylation of dark regions within specific Alzheimer's disease risk genes and repetitive elements, was observed across disease stages.
3. **Future directions**: Our study provides a basis for future work focusing on genomic and epigenomic changes in the dark regions, the centro/pericentromere and rDNA in the context of Alzheimer's disease pathology.

### 2.2 DNA processing and read alignment

Approximately 80 mg of tissue was homogenized with a Dounce and centrifuged through a sucrose gradient to isolate nuclei. High molecular weight DNA was extracted from nuclear isolates using a standard phenol chloroform protocol. Library preparation was performed by the University of California Davis genomics core using the SQK‐LSK 110 kit (ONT) with shearing to obtain 10 kbp length reads. Libraries were then multiplexed, randomized into groups of three per PromethION flow cell, and sequenced to obtain a desired target coverage of 10X per sample. Reads were basecalled with Guppy version 4.0.11 (ONT). Nanopore‐derived reads were aligned to the GRCh38 genome (University of California at Santa Cruz [UCSC]) and the HS1‐CHM13 genome using MiniMap2^30^ using nanopore sequencing‐specific parameters to obtain bam files. Mosdepth^31^ was used to determine the average coverage per sample. An additional 30X coverage NeuN‐sorted nanopore dataset for Alzheimer's disease (*n* = 10, Braak 4.6–6) and controls (*n* = 10, Braak 1–2.5)^28^ was downloaded from the National Center for Biotechnology Information Sequence Read Archive (NCBI SRA) repository (PRJNA636606) and was subject to the same post‐processing steps.

### 2.3 Non‐reference retrotransposon insertion calling

All bam files were analyzed simultaneously with TLDR (default parameters) to reduce false negative calls in any single sample. This resulted in a retrotransposon insertion file with calls across all 18 samples. Insertions called by TLDR were then filtered to retain insertions with a target site duplication (TSD), an UnmapCover (fraction of inserted sequence covered by retrotransposon sequence) value of 0.8 or more, an intact 3′ end of the retrotransposon of interest and a PASS quality score to eliminate low confidence/quality insertions. We defined a putative L1 endonuclease (L1en)‐mediated singleton in a post‐mitotic cell as a call that was only supported by a single insertion‐spanning read of 10 kbp or longer in a single individual who had at least 10 additional reads across the region, all of which lack the insertion, along with a TSD greater or equal to 5 bp and less than or equal to 31 bp and an L1en motif at the insertion site. All singleton insertion alignments were further visualized with the Integrative Genomics Viewer (IGV) to confirm L1en insertion characteristics.^32^ The NeuN‐sorted dataset was analyzed similarly.

### 2.4 NAHR analyses

Nanopore‐derived reads were aligned to both the GRCh38 and CHM13 genomes using the LAST alignment software with parameters suggested by the TE‐reX pipeline.^28^, ^33^ NAHR events were then called using TE‐reX^28^ and recommended analysis pipeline to obtain singleton and polymorphic events. Events were only retained if they had a value equal to or less than 1E‐4 and were identified as a singleton event. NAHR enrichment values were calculated by dividing the genome into 100 kbp windows and obtaining the ratio of NAHR events to the total repetitive elements per window.^28^

### 2.5 Structural variant calling

Individual bam files were subjected to structural variant calling using SNIFFLES2^34^ (default parameters) and subsequently combined into a multi‐sample variant call file with the SNIFFLES2 multi‐sample calling module. The variant call file was then filtered to retain variants 50 bp or larger. The locations and potential effects of the structural variants in the GRCh38 were determined using snpEFF. Insertion nucleotide sequences were extracted from the variant file and searched against the NCBI nucleotide and RefSeq^35^ databases using BLASTn^36^ (default parameters) within the Galaxy platform.^37^ Hits were retained if the query insertion was covered by 50% or more of a subject sequence in the database. To compare structural variants across Braak groups, we required that the region of a given structural variant was genotyped across all 18 samples. A variant that was only found in samples belonging to a single Braak group was considered unique to that group.

### 2.6 Normalization and statistical comparison of non‐reference retrotransposon insertions and structural variants

TLDR insertions, TE‐reX NAHR events, and SNIFFLES2 variants unique to a Braak group were compared via ANOVA. To account for differences in sequencing coverage, insertion counts were normalized by dividing the raw count value by the total number of diploid human genomes sequenced (Gbp sequenced divided by 6.4 Gbp).

### 2.7 Characterization of retrotransposon insertions and structural variants

Coordinates from the TLDR, TE‐reX, and SNIFFLES2 output were processed with the R GenomicRanges package.^38^ Overlap of insertions and variants with regions (genic/non‐genic, dark regions, tandem repeat regions) were determined with AnnotatR.^39^ Bedtools^40^ was used to identify variants near single nucleotide polymorphisms. Gene graphs were first generated by IGV^32^ and then modified in Adobe Illustrator for clarity. L1Hs subfamilies were identified by aligning L1Hs loci to the L1.2 (active LINE‐1: GenBank accession number M80343) and identifying the L1‐Ta (L1Hs‐Ta) diagnostic ACA and G nucleotides in the 3′ untranslated region (UTR). HERV‐K open reading frames were determined by aligning HERV‐Ks of interest to the consensus HERV‐K sequence (DFAM ID: DF0000188). HERV‐K non‐reference insertions were manually analyzed for coding potential and completeness compared to the full‐length HERV‐K consensus sequence. To identify other HERV insertions, SNIFFLES2‐identified insertions were searched against a custom HERV internal and LTR5 database with sequences from RepBase^41^ using BLASTn^36^ (default parameters) within the Galaxy platform.

### 2.8 CpG 5mC methylation analysis

The 5mC modifications were called using the Nanopolish^42^ call‐methylation pipeline with single bam files described above on both the GRCh38 and CHM13 genomes. Alignments with a mapping quality score of 20 or more were included in calculating log‐likelihood methylation ratios. Nanopolish‐generated log‐likelihood ratio values were then converted to methylation frequencies with accompanying Nanopolish scripts. The default log‐likelihood ratio threshold suggested by the authors was used to filter out low‐quality calls. Samples were then analyzed via multidimensional scaling (MDS) to visualize sample clustering. Sample CTRL‐1 had a previous cancer diagnosis and was identified as an outlier; this sample was removed from further analysis. Average methylation frequencies across all samples were annotated as within genes, CpG islands or repeat regions (defined by UCSC RepeatMasker) using annotatR. Methylation graphs of genomic loci were created with methylArtist,^43^ methylkit,^44^ and NanomethViz.^45^

### 2.9 Differential methylation analysis

Differentially methylated CpG sites were identified using DSS,^46^ a tool that models methylation distributions with a beta‐binomial model to compute changes in methylation at the loci and regional level, taking sequencing depth into account. As Nanopolish groups CpGs within 10 bp of each other into a single methylation call, we only assigned a methylation value to the most 5′ CpG within a given cluster to avoid artificial inflation of depth. Individual differentially methylated CpG sites were considered significant if they reached a false discovery rate (FDR) of 0.05 or less for both the GRCh38 and CHM13 genomes. DSS was then used to identify and aggregate significant neighboring CpG sites (uncorrected *p* value less than 0.05) into differentially methylated regions (DMRs) compared to the control.^47^ AreaStat, which represents the sum of test statistics for CpG sites within a DMR, was calculated for Braak III vs. Braak 0 and Braak V/VI vs. Braak 0. AreaStat allows the ranking of DMRs, with higher values indicating a higher likelihood that the CpG is differentially methylated. A sample CpG site methylation frequency was only included if the call was supported by three or more Nanopolish‐called sites. Comparisons were made for Braak III vs. Braak 0 and Braak V/VI vs. Braak 0. Alu elements and LTR5Hs with the largest changes in methylation between groups were then manually inspected using the GRCh38 GeneHancer Regulatory Elements and the Gene Interaction table^48^ within the UCSC genome browser^49^ to determine if any overlap occurred. For inclusion in methylation average comparisons of retrotransposons *en masse*, individual loci were required to have 10 or more called CpG sites. To exclude highly fragmented retrotransposon loci, a length threshold depending on the type of retrotransposon was implemented: HERV‐K (> 6000 bp), LTR5_Hs/A/B (> 900 bp), LINE1‐Hs (> 5900 bp), AluYa5/Yb8 (> 280 bp), and SVA‐E/F (> 1000 bp).^43^ Other repetitive elements such as centromeric satellite regions were required to have 10 or more CpG sites called within a given region. A promoter region was only counted once in each direction even if multiple DMRs fell within the promoter region.

### 2.10 Metachromosome analysis

Metachromosomes were generated using a custom R script following previously described methods.^50^, ^51^ First, a bed file was created with Bedtools^40^ to obtain 100 kb windows across the CHM13 genome (excluding sex chromosomes and mitochondria). GRange objects^38^ were then created for centromere regions, cytoband information, and biological values to plot DMR or insertion coordinates. The overlap of centromeres, cytobands, and genomic windows was then determined by ranking each genomic window based on its chromosome arm and position to standardize distances from the p arm telomere (rank = 1) to the centromere (rank = 0) and q arm telomere (rank = ‐1) to the centromere. Windows that fully overlapped with the active alpha satellite region of the centromere were assigned a rank value of zero. For plotting of DMR centromere and non‐centromere ranges, a rolling average of methylation changes was calculated per window over an 11‐window span (∼1 Mb) for both centromere and non‐centromere ranges. Results were combined and normalized by segmenting chromosome positions into 500 meta‐bins ranging from 0 (q arm telomere) to 500 (p arm telomere), allowing for the grouping of chromosomal windows across chromosomes of different lengths. For each meta‐bin group, the average methylation change was calculated and plotted across the bins. For each meta‐bin group, a line plot for each chromosome shows the average methylation change across meta‐bins, marking centromeric (bins 249–251) and telomeric boundaries (bins 0‐1 and 499–500), and overlaying reference lines for average DMR. The same pipeline was followed for analysis of singleton insertions in Braak 0, Braak III or Braak V/VI, except that raw counts were taken per window rather than rolling averages. The average insertion count per bin was then calculated and normalized to the mean insertion count per window (excluding windows with no insertions).

### 2.11 Enrichment analysis and statistical tests

A permutation test (regioneR function permTest^52^) was used to determine if there were more differentially methylated regions within centromeres and rDNA arrays than what would be expected by chance. All graphs were generated with R packages ggplot2,^53^ TreeMap or adapted from IGV.^32^ Lola^54^ was used for genomic region enrichment with UCSC datasets. KaryotypeR was used for karyotype graphs.^55^ Gene lists for promoter methylation analyses were analyzed for KEGG pathway^56^ enrichment using ClusterProfiler.^57^ One‐way analysis of variance (ANOVA) and Student's t‐test were performed in base R or with the ggstatsplot^58^ R package.

## 3 RESULTS

### 3.1 Nanopore long‐read sequencing of DNA extracted from aged human brain

DNA was extracted from isolated nuclei of *post mortem* frontal cortex of six individuals at Braak 0, six individuals at Braak stage III and six individuals at Braak stage V/VI with a clinical diagnosis of Alzheimer's disease (Table S1). The 18 human frontal cortex samples utilized in this study had an average age of 76.6 years (Table 1). Sequencing was performed using the Oxford Nanopore Technologies PromethION sequencing platform. We obtained a mean read quality score of 11, with a mean sample N50 of 23.2 kbp and an average genomic coverage of 7.38X (Table S2). ANOVA testing reveals no statistically significant difference among Braak 0, III, and V/VI for *post mortem* interval (PMI), total bases sequenced, read quality, read length or genome coverage. While the age of individuals at Braak III or Braak V/VI do not significantly differ from Braak 0, post‐hoc *t*‐test indicates that the age of individuals at Braak III is significantly higher than those at Braak V/VI.

**TABLE 1:** Brain demographics and sequencing metrics

Parameter	Braak 0	Braak III	Braak V/VI	All	Range	*p*‐Value
Total # sequenced	6	6	6	18		
Age	76 years	82.3 years	71.3 years	76.6 years	67–92 years	0.03^*^, Braak III vs. V/VI
% Female	50%	50%	33%	44%		
*Post mortem* interval	10.33 h	14.67 h	8.75 h	11.89 h	5–24 h	0.63

	Mean	Range	*p*‐Value			
Total bases sequenced	25.5 Gbp	14.2–41.6 Gbp	0.66			
Read quality	11.7	11.5–11.9	0.21			
Read length N50 (kbp)	23.2 kbp	9.66–31.58 kbp	0.66			
Genome coverage	7.4X	4.1X–12.1X	0.65			

*Note*: Sample characteristics and associated sequencing statistics.

* Represents the *p* value obtained from the post hoc *t‐*test.

### 3.2 Detection of retrotransposon singleton insertions in the aged human brain

We first utilized our long‐read sequencing data across all 18 human brain samples to identify retrotransposon sequences that are not captured in the new CHM13 human telomere‐to‐telomere genome (File S1). TLDR, ^43^ a tool designed to detect retrotransposon insertions using long‐read sequencing data, identified 642 retrotransposon sequences that were unique to a single sample and are lacking in the CHM13 reference genome (Figure S1A). These non‐reference genome retrotransposons may result from retrotransposition events that occurred in an individual in utero or during life or could represent retrotransposons that are polymorphic within the human genome that the individuals used to generate reference genomes happened to lack.

**FIGURE 1:**
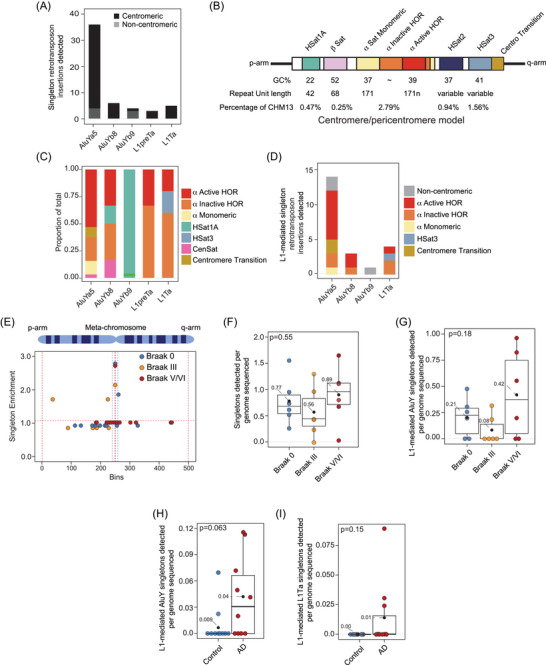
Enrichment of putative singleton retrotransposon insertions in the centromere of the aged human brain. (A) Total counts of all singleton retrotransposon insertions by subfamily grouped by whether they are present in the centromere. (B) Graphic model of centromeric and pericentromeric regions. (C) Barplot of the fraction of singleton insertions detected within functional centromeric repeats. (D) Total counts of all L1en‐mediated singleton retrotransposon insertions by subfamily, grouped by centromeric repeat. (E) Mean count of retrotransposon singleton insertions detected across all autosomes, normalized for chromosome length plotted across a metachromosome. (F) Boxplots of singleton retrotransposon insertion counts normalized to sequencing depth. (G) Boxplots of putative L1en‐like mediated singleton AluY insertion counts normalized to sequencing depth. (H) Boxplots of NeuN‐sorted putative L1en ‐like mediated singleton AluY insertion counts normalized to sequencing depth. (I) Boxplots of NeuN‐sorted putative L1en ‐like mediated singleton L1Hs insertion counts normalized to sequencing depth. Statistical analysis was done via analysis of variance analysis for F and G, and a Student's *t‐*test for H and I. Black dots on boxplots represent the group mean normalized insertion value.

We next identified potential somatic retrotransposition events marked by singleton insertions (insertions only supported by a single read) following methods similar to Siudeja and colleagues.^59^ As the site of L1‐mediated retrotransposon insertion is random, de novo insertions should be present as (1) singletons if they occur in post‐mitotic cells, or (2) low frequency variants if they occur in dividing cells. Of all non‐reference insertions unique to a given individual, we identified 54 as singletons based on presence of at least ten additional reads covering the location of the insertion, all of which lacked the insertion, and presence of TSD‐like flanking sequences indicative of L1‐mediated transposition (File S2). Intact TSDs are a signature of a recent target‐primed reverse transcription event that consists of a duplication of a short DNA sequence near the target site (canonically AA/TTTT) of insertion on either side of the inserted element.^60^, ^61^, ^62^, ^63^, ^64^ Among retrotransposon families, the Alu subfamily of SINE elements was the most abundant class of TSD‐harboring singleton insertions among our human brain samples; AluYa5 was the most active member within the Alu subfamily (Figure 1A). Interestingly, the majority of singleton insertions (unlike non‐singleton insertions) were located in centromeric and pericentromeric regions of the genome, and predominantly affected chromosomes 11, 14, 16, and 20 (Figure S1B, File S2). Further analysis of singleton Alu insertions with respect to centromeric repeats (Figure 1B) revealed that they were predominantly located in alpha satellite active highly ordered repeat (HOR) regions, while singleton L1 insertions were more frequent in inactive HORs (Figure 1C).

As some LINE and SINE retrotransposons are transpositionally active in somatic cells, including postmitotic neurons,^65^, ^66^, ^67^, ^68^ we used previously established criteria^43^, ^69^, ^70^ to characterize L1 endonuclease (L1en)‐mediated insertion events from evolutionarily “young” elements. After hand‐filtering our list of singleton insertion events to include reads of 10 kbp or longer that harbor characteristics of L1en‐mediated transposition, including presence of a polyA tail, an L1en motif at the predicted insertion location and manual inspection of insertions using IGV,^32^ we detected 22 putative L1en singleton insertions across the 18 brains analyzed, most of which belong to the AluYa5 subfamily (Figure 1D). While most detected Alu insertions were intact, we found that L1en‐mediated L1Hs singleton insertions were severely truncated at the 5′ end (File S2), consistent with a 3′ end bias of insertion.^71^ While we attempted to determine the genomic source of L1Hs singleton insertions via Blat alignment of the insertion sequences against the CHM13 genome, alignments were ambiguous due to lack of transduction sequences. We observed significant enrichment (*p* value of 1E‐15, Fisher's exact test) of L1en‐mediated insertions in centromeric regions of the genome, with 17 occurring in HOR segments of the centromere.

To assess retrotransposition in brains affected by Alzheimer's disease, we next compared overall singleton insertions and L1en‐mediated singleton insertions in brains at Braak III and IV/V versus controls. All three groups featured enrichment of singleton insertions within the centromere when plotted across a metachromosome (Figure 1E). While abundance of general singleton insertions did not differ among Braak stages (Figure 1F), restricting our analysis to L1en‐mediated singletons revealed a trending increase (*p* value of 0.18, total of 18 insertions) of AluY, but not L1Hs (*p* value of 0.92, total of 4 insertions) insertions in brains with the highest degree of tau burden (Figure 1G, Figure S1C).

To determine if somatic retrotransposition occurs specifically in neurons affected by Alzheimer's disease, we analyzed nanopore long‐read DNA sequencing data of NeuN‐sorted neuronal nuclei from a publicly available Alzheimer's disease dataset with 30X sequencing coverage^28^ (File S2). Increased coverage and analysis specifically in neurons revealed a stronger trend of increased AluY retrotransposition in Alzheimer's disease compared to control (*p* value of 0.063, total of 24 insertions) (Figure 1H). While potentially somatic insertions of L1Hs did not significantly differ between Alzheimer's disease cases vs. controls in neurons (p value of 0.15, total of three insertions), L1Hs singletons were only detected in samples affected by Alzheimer's disease (Figure 1I). L1en‐mediated singleton insertions were only detected in the centromere/pericentromere (File S2). While centromeric/pericentromeric singletons had relative low mapping quality (MAPQ) scores of one, the absence of L1en‐mediated singletons in many samples suggests that detection of such insertions is not simply a technical artifact. Mean estimated L1en‐mediated singleton insertion rates for 7X coverage bulk sequencing and 30X coverage neuronal datasets were 0.74 and 0.0245 per cell, respectively. Taken together, our analyses suggest that specific AluY and LINE‐1 family members are mobile in cells of the aged human brain and/or their precursor cells, that *de novo* insertion events are rare and occur more frequently in the centromere/pericentromere, and that the AluY element may mobilize to a greater extent in brains affected by Alzheimer's disease.

### 3.3 NAHR between repetitive elements in the aged human brain

Repetitive element‐derived structural variants can arise from NAHR events between homologous high‐copy repeats in the genome.^72^ We utilized TE‐reX,^28^ a tool developed to detect NAHR events between transposable elements, to identify singleton retrotransposon‐associated NAHR events within our set of 18 human brains. We utilized CHM13 and GRCh38 human reference genomes to leverage their respective strengths: assembly completeness (CHM13) vs. more thorough annotation (GRCh38).

**FIGURE 2:**
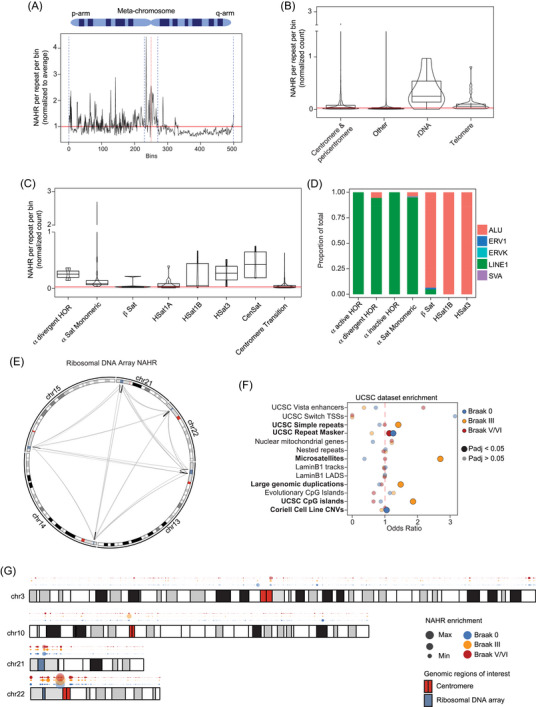
NAHR in the aged human brain. (A) Metachromosome of the rolling mean of NAHR enrichment values across all autosomes, normalized for chromosome length. (B) Boxplots of singleton NAHR enrichment values for different chromosomal structures. (C) Boxplots of singleton NAHR enrichment values for different centromeric repeat structures. (D) Barplot of transposable element‐associated NAHR events among different centromeric repeat structures. (E) Circular karyotype map depicting interchromosomal (gray) and intrachromosomal (black) NAHR between rDNA arrays. (F) Dotplot of NAHR event enrichment on different UCSC genomic sets based on Braak stage. (G) Karyotype map depicting hotspots of NAHR based on Braak stage. Red and blue rectangles within chromosomes represent centromeres and rDNA arrays, respectively. Red lines in A, B, C indicate the average NAHR value across the genome. NAHR, non‐allelic homologous recombination; rDNA, ribosomal DNA; UCSC, University of California at Santa Cruz.

We first identified recombination hotspots from a total of 84,280 putative singleton events across the CHM13 genome by counting singleton NAHR events within 0.1 Mbp windows and normalizing counts by the number of repetitive elements present within the window.^28^ To obtain a genome‐wide view of NAHR, enrichment values were plotted along a metachromosome, which revealed that centromeric/pericentromeric regions exhibited the highest normalized enrichment values (Figure 2A), consistent with previous work.^28^ We then determined the distribution of NAHR enrichment values for large chromosomal regions, which revealed increased recombination activity primarily in acrocentric chromosome arms, rDNA arrays, and centromeric/pericentromeric regions compared to other genomic areas (Figure 2B, Figure S2A). Most detected recombination events were predicted to result in interchromosomal rearrangements mediated by SINEs (Figure S2B, C), consistent with previous findings in neurons isolated from *post mortem* human brain.^28^

Given the high enrichment of NAHR in centromeric and pericentromeric regions, we analyzed NAHR within the functional repeat arrays that compose these regions. NAHR events were detected in HOR repeats (monomeric and divergent), classical human satellite regions, beta satellites, and other centromeric repeat regions (Figure 2C). Interestingly, we found that NAHR within HORs were composed primarily of L1‐L1 recombination events, while NAHR within beta and classical human satellites reflected recombination between Alu elements (Figure 2D). We also detected unique individual regions of enrichment in many peritelomeric and telomeric regions such as the telomeric region of chromosome 17, as well as intergenic segmental duplications such as those present in chromosomes 4 and 6 (File S3).

Strikingly, 47S rDNA arrays were found to have higher median recombination enrichment compared to other NAHR‐prone regions such as telomeres or the centromere. While the majority of detected NAHR rDNA events occurred between different chromosomes, we also detected intrachromosomal NAHR events measuring 35 kbp, 45 kbp, and 95 kbp in length (Figure 2E, Figure S2D) that reflect 47S rDNA that has been deleted, inverted, or duplicated. While insertion and inversion NAHR rDNA events were marked by Alu‐Alu recombination, deletion events appeared to stem primarily from LINE‐LINE NAHR events, with additional contribution of rRNA‐HSA repeats (File S3).

While the overall frequency of repeat‐associated NAHR events did not significantly differ among Braak stages (Figure S3A‐O), varying values of NAHR enrichment could be discerned in distinct regions of the genome based on Braak stage (examples provided in Figure 2F, G, full karyotype maps provided in Figure S4A). UCSC dataset enrichment analysis further revealed significant changes in NAHR localization (112,108 singleton events) to genomic regions consisting of simple repeats, microsatellites, CpG islands, and annotated segmental duplications at Braak III (Figure 2F, G).

### 3.4 Structural variants in the aged human brain

We next leveraged our dataset to identify complex, likely germline‐derived repetitive structural variants in the human brain using the more thoroughly annotated GRCh38 genome (Figure S5A, File S4). Structural variants were most frequently detected in introns and exons (Figure S5B). Length distribution of structural variants was highly variable, with insertions having a larger median length compared to other variants (Figure S5C). We next utilized SNPeff^73^ to predict how these variants may affect gene function. Most structural variants were classified as modifier variants in non‐coding genes/intergenic regions or moderate (non‐disruptive) impact variants (Figure S5D). We detected structural variants in genes associated with neurodegeneration, including an intronic deletion/inversion in *APP*, a tandem duplication in *ATXN1*,^74^ and a deletion in *MAPT* (File S5).

We next identified non‐reference structural variants that fell within the 6054 dark regions of the human GRCh38 reference genome.^4^ Among the 18 brains analyzed, we detected 1123 insertions in the dark regions of 787 genes, 60 of which occurred in dark regions of exons. 1781 deletions were associated with dark regions among the 18 brains analyzed. Interestingly, 23 Alzheimer's disease risk genes contained dark regions with previously‐undocumented structural variants, some of which were in close proximity to single nucleotide polymorphisms (SNPs) defined as risk variants for neurodegenerative diseases based on the Alzheimer's Disease Sequencing Project (ADSP)^75^ or the European Bioinformatics Institute genome wide association study (EFO ID: EFO_0005772). For example, three dark region‐associated insertions and a retrotransposon insertion were detected near or overlapping Alzheimer's disease‐associated SNPs within *ATP binding cassette subfamily A member 7* (*ABCA7*) (Figure S5E), which encodes an ABC transporter that regulates lipid metabolism,^76^, ^77^ amyloid processing, and clearance.^78^, ^79^, ^80^ A deletion and an insertion were detected within a dark region of an intronic SVA retrotransposon within vasoactive intestinal peptide receptor 2 (*VIPR2*), near an Alzheimer's disease‐associated SNP overlapping a deletion present in 10 out of 18 brains analyzed (Figure S5F). Similarly, most brains analyzed harbored a deletion overlapping a dark intronic AluYa5 element within chromodomain helicase DNA binding protein 2 (*CHD2*) in close proximity to an Alzheimer's disease‐associated SNP (Figure S5G, File S5). Dark region insertions were also detected near SNPs associated with amyotrophic lateral sclerosis, multiple system atrophy, multiple sclerosis, and spinocerebellar ataxia. Many non‐reference insertions and deletions within dark regions were shared among all samples, likely reflecting common circulating variants, while others were only found in single samples, reflecting less common circulating variants, new variants that occurred in the germline, or variants of somatic origin.

We next identified structural variants unique to Braak 0, Braak III or Braak V/VI samples using the CHM13 and GRCh38 reference genomes. We required that the given locus was genotyped in all 18 samples to ensure each variant was unique to a particular group. The number of structural variants detected per sample did not significantly differ between Braak 0, Braak III, and Braak V/VI brains (Figure S6A, B), nor did structural variant length (Figure S6C). These results also hold true when Braak III and V/VI were combined into one group and compared to Braak 0. Despite the lack of change between Braak stages among the small number of brains analyzed, the overlap of structural variants within dark regions of genes associated with human disease highlights the value of approaches that resolve complex regions of the genome and identifies new variants that can be specifically targeted for analysis in a larger cohort of human samples.

#### 3.4.1 DNA methylation in the aged human brain

We next analyzed 5mC DNA methylation using the CHM13 and GRCh38 genomes. A sample from an individual with a previous cancer diagnosis was an outlier in terms of 5mC methylation and was thus removed from subsequent analysis (Figure S7A). We focused on CpG sites with a 5mC call in at least three samples, resulting in 24.8 million and 23 million CpG sites for analysis based on the CHM13 and GRCh38 genomes, respectively.

**FIGURE 3:**
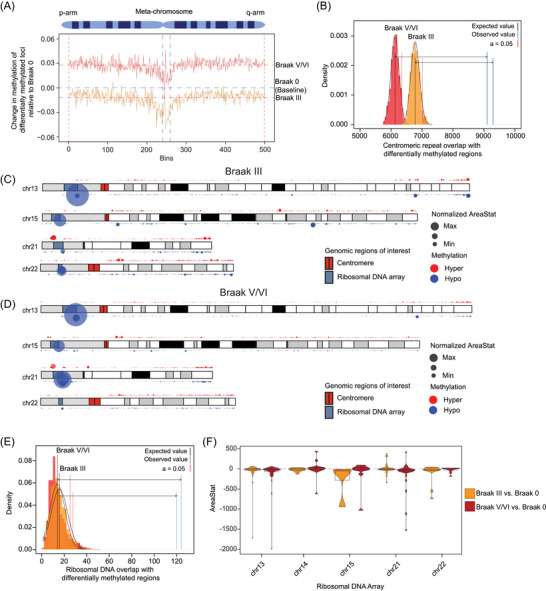
DNA methylation in brains affected by Alzheimer's disease. (A) Metachromosome of the rolling mean of the difference in methylation values of differentially methylated regions across all autosomes and normalized for chromosome length for Braak III vs. Braak 0 and Braak V/VI vs. Braak 0. Orange and red horizontal dashed lines represent the average change across the metachromosome for Braak III and Braak V/VI, respectively. The blue horizontal dashed line represents the baseline value of Braak 0. (B) Permutation test of differentially methylated regions localized to the centromere in Braak III vs. Braak 0 and Braak V/VI vs. Braak 0, *p* = 0.0001. (C, D) Karyotype of example chromosomes with significant changes in methylation in Braak III and Braak V/VI. Red and blue rectangles within chromosomes represent centromeres and rDNA arrays, respectively. (E) Permutation test of differentially methylated regions localized to rDNA arrays in Braak III vs. Braak 0 and Braak V/VI vs. Braak 0, *p* = 0.0001. (F) Box/violin plot of areaStat values for differentially methylated regions between Braak III vs. Braak 0 and Braak V/VI vs. Braak 0. rDNA, ribosomal DNA.

We used the DDS callDMR function to leverage single‐nucleotide CpG resolution and the documented increase in detection power when combining differentially methylated CpG sites. Changes in uncorrected DMRs across the CHM13 genome were analyzed for Braak III vs. Braak 0 (93,441 DMRs) and Braak V/VI vs. Braak 0 (88,423 DMRs) (Figure S7B, C, Files S6, S7), and the rolling average of the change in methylation of statistically significant DMRs was plotted across a metachromosome (excluding sex chromosomes) for each comparison. While the average change in methylation was decreased in Braak III and elevated in Braak V/VI compared to Braak 0 (Figure 3A), some regions shared a conserved directional change in Braak III and Braak V/VI compared to Braak 0. Centromeric/pericentromeric regions, for example, were demethylated at Braak III and V compared to Braak 0, albeit to varying degrees. Indeed, a permutation test revealed that centromeric methylation was significantly dysregulated in tau‐affected brains compared to control (Figure 3B, Figures S7D‐H).

The DSS area statistic (areaStat), which sums the test statistics for CpG sites within a DMR, was calculated for Braak III vs. Braak 0 and Braak V/VI vs. Braak 0, taking into account both the length and the number of CpG sites with differential methylation.^46^ A larger area suggests a higher probability that the region is a genuine DMR. We determined the distribution of DMRs across autosomes in Braak III and Braak V/VI cases by plotting areaStat of DMRs on a karyotype graph (Figure S8A, B; Figure 3C, D include examples within chromosomes 13, 15, 21, and 22). We identified many DMRs that are shared between early‐ and late‐stage disease based on magnitudes of change in the same or opposite direction, some of which overlapped with rDNA array model regions of the CHM13 genome. Indeed, a permutation test revealed that 47S rDNA regions were significantly enriched for DMRs in Braak III and Braak V/VI compared to Braak 0 (Figure 3E). DMRs overlapped some but not all 47S rDNA copies across all five rDNA array regions, with the largest magnitude of disease‐associated change being hypomethylation.

We next analyzed 5mC changes within promoters across Braak stages using the GRCh38 genome. We detected differential methylation of 20,352 unique promoters of 7852 genes at Braak III compared to Braak 0, while 19,638 promoters of 5765 genes were differentially methylated at Braak V/VI (File S8). We observed a subtle change in the bimodal distribution of 5mC modifications in differentially methylated promoters (Figure S9A, B), in line with promoter element MDS analysis.^81^ DMRs in brains at Braak III had higher densities at 0.25 and 0.75 compared to Braak 0, while brains at Braak V/VI shift towards no methylation (0) or complete methylation (1). While promoter methylation differs among brains at Braak 0, Braak III, and Braak V/VI, we identified over 7072 hypomethylated promoters that were unique to brains at Braak III related to KEGG pathways^56^ related to phospholipase D, FC gamma receptor mediated phagocytosis, gonadotropin‐releasing hormone (GnRH) signaling pathway, Rap1 signaling pathway and actin cytoskeleton regulation (Figure S9C). The ∼3000 hypermethylated promoters that were shared between Braak III and Braak V/VI brains related to pathways involving calcium and oxytocin signaling, focal adhesions, phospholipase D and glutamatergic synapses (Figure S9D).

We next focused on DNA methylation within “dark” regions of the genome to fully leverage our long‐read approach. We detected 1150 and 1333 DMRs at Braak III vs. Braak 0, and Braak V/VI vs. Braak 0, respectively. Four DMRs in Braak III vs. Braak 0 fell within dark regions of Alzheimer's disease risk genes (*ABACA7*, *AMY1A*, *CHRFAM7A*, *CR1*). DMRs within *AMY1A*, *CHRFAM7A*, and *CR1* were also present at Braak V/VI (File S8). 29 5′ UTRs were differentially methylated at Braak III; 37 5′ UTRs were differentially methylated at Braak V/VI, including hypomethylation of Alzheimer's disease risk genes *AMY1A* and *FMR1* within dark regions in their 5′ UTR at Braak V/VI.

#### 3.4.2 DNA methylation within transposable element sequences of the human brain

Consistent with prior observations,^82^, ^83^, ^84^ we found that DNA methylation levels correlated with the evolutionary age of retrotransposons, with younger elements exhibiting higher methylation frequencies than older elements. With the exception of human endogenous retrovirus (HERV)‐K elements, retrotransposons were found to be heavily methylated in the aged human brain, with a mean methylation value exceeding 0.75 (Figure S10A‐E). Young Alu and LINE retrotransposon family members had generally higher levels of 5mC compared to older Alu and LINE family members, perhaps reflective of an increased need to repress their transpositional capacities. HERV families and other long terminal repeat (LTR) retrotransposons featured less methylation of younger elements.

While previous studies report widespread changes in 5mC at different stages of Alzheimer's disease in the frontal cortex,^85^ methylation within dark regions has not been studied in brains of patients with Alzheimer's disease. An MDS plot of CpG methylation frequency in autosomal repetitive elements revealed no clustering based on sex or Braak stage (Figure S11A). While we did not detect differences in 5mC methylation of retrotransposon subfamilies between Braak stages when retrotransposons were grouped *en masse*, similar to previous studies in the blood of individuals with Alzheimer's disease,^86^, ^87^ methylation within repetitive regions was clearly dysregulated at the level of individual retrotransposon loci (Figure S11B‐H). Distribution of 5mC modification in differentially methylated repetitive elements shifted in opposite directions according to Braak stage, with generally less methylation at Braak III and generally increased methylation at Braak V/VI (Figure S11B).

**FIGURE 4:**
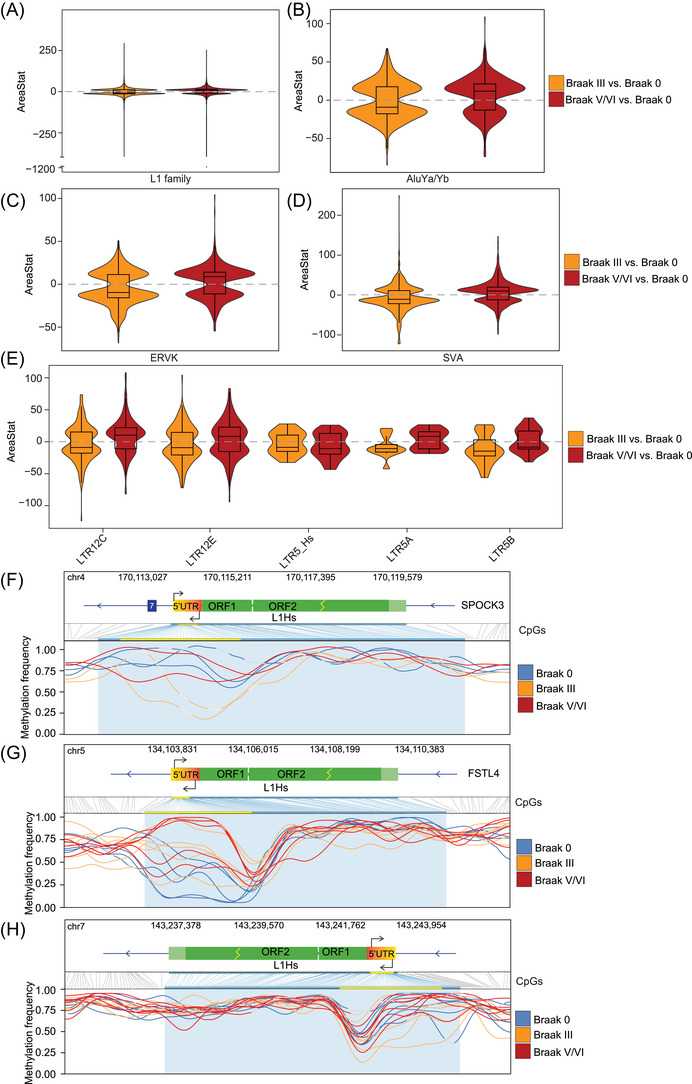
DNA methylation patterns of retrotransposons in Alzheimer's disease. Box/violin plots of areaStat values for DMRs between Braak III vs. Braak 0 (orange) and Braak V/VI vs. Braak 0 (red) for (A) LINE‐1 family (B) AluYa/Yb, (C) HERV‐K subfamily, (D) SVA subfamily, (E) and LTR sequences. (F, G, H) Methylation frequency of CpG sites across the indicated genomic locus per sample, colored by Braak group. Statistically significant changes in CpG methylation detected in promoter regions are highlighted in yellow; yellow lightning bolts denote truncated ORFs. Lighter colors within lines in F and H indicate gaps in sequencing. DMR, differentially methylated region; LTR, long terminal repeat; ORF, open reading frame.

We analyzed the methylation areaStat for select families and subfamilies of retrotransposons to more precisely quantify the degree of differential methylation. We observed changes in areaStat for L1 elements (with some loci accounting for large magnitude changes), with most of the more significant changes reflective of hypomethylation (Figure 4A). We then separated the L1 family into L1Hs (young) and ancestor‐shared (old) L1 subfamilies; distribution of L1Hs and ancestral L1 subfamily methylation was similar among Braak stages (Figure S11C). We observed similar changes in methylation areaStat for AluYa/Yb, HERV‐K, and SVA subfamilies (Figure 4B‐D). Given that LTR elements can function as alternative promoters for genes and as promoters for HERVs, we analyzed methylation patterns of the LTR12 and LTR5 families. Within the LTR12 family, LTR12C exhibited the most pronounced changes in methylation of specific loci compared to LTR12E and other LTR subfamilies. Similarly, the younger LTR5 family demonstrated milder, though significant, methylation changes compared to LTR12 subfamilies (Figure 4E). We also detected differential methylation in several solo LTR5Hs and Alu elements that are predicted to be regulatory regions based on the GeneHancer database (Table S3).

We then analyzed changes in methylation of selected differentially methylated non‐fragmented (> 5900 bp) L1Hs retrotransposons on a per‐sample basis. Four L1Hs loci (Figure S12A‐E) featured demethylation at the 5′ UTR, suggesting that these elements may be source loci for L1Hs transcripts in the human brain. When comparing among Braak stages, we found statistically significant hypomethylation in the promoter of individual L1Hs loci such as an intergenic L1‐Ta (transcriptionally active) (7q34) and an intronic L1‐Ta in *SPOCK3* (4q32.3) at Braak III compared to Braak 0 (Figure 4F, G, File S9). Our analysis also revealed examples of LINE hypermethylation at Braak III (Figure 4H). We detected promoter demethylation of an intact L1‐Ta element promoter,^43^ regardless of Braak stage, that is reported to be a source of somatic retrotransposition in the developing hippocampus (Figure S12C).^20^, ^88^ Several LTR5Hs loci, in both provirus and solo forms, were differentially methylated at Braak III and/or Braak V/VI compared to Braak 0 (Figure S13A, B, File S9), with some hypomethylated 5′ LTR5Hs loci adjacent to proviral HERV‐K elements. For example, the 5′ LTR5Hs of HERV‐K107 (7p22.1) was found to be modestly hypomethylated in Braak III and Braak V/VI and has been previously implicated in the pathogenesis of human diseases.^89^, ^90^, ^91^ Overall, our analyses provide novel insights into methylation of individual retroelements and highlight the importance of analyzing such elements at the locus level rather than *en masse*.

## 4 DISCUSSION

While previous studies have identified risk variants and epigenetic dysregulation in Alzheimer's disease,^1^, ^92^, ^93^ technical limitations result in exclusion of many dark/repetitive regions from analysis. Long‐read sequencing, associated bioinformatic tools, and a more complete human genome assembly help overcome these challenges. Nanopore sequencing‐based analysis of retrotransposon insertions, retrotransposon‐associated NAHR, structural variants, and 5mC modification in *post mortem* human Alzheimer's disease brains revealed genomic and epigenomic changes that concentrate in retrotransposon‐rich centro/pericentromeric regions and rDNA arrays. Our findings build upon a long‐read sequencing study of DNA isolated from neuronal nuclei of control and late‐stage Alzheimer's disease brains.^28^ Inclusion of Braak III tissue allowed us to capture changes occurring in early disease; leveraging the telomere‐to‐telomere reference genome and methylation data allowed us to comprehensively interrogate genomic dark regions.

Long‐read sequencing‐based quantification of L1en‐mediated retrotransposition events in the aged brain revealed insertion rates of 0.145 and 0.0147 per cell for 7X coverage bulk sequencing and 30X coverage neuronal sequencing, respectively. This rate of insertion is higher than L1Hs estimates based on whole genome amplification^20^, ^21^, ^94^ but less than estimates of 13.7 per neuron and 6.5 per glia based on retrotransposon capture sequencing.^22^ We note that our estimations include L1Hs and Alu elements, whereas previous studies focused only on L1Hs. We find that AluYa5 is the most transpositionally active retrotransposon in the human brain, consistent with data from the 1000 genomes project.^95^ In line with previous research indicating that centromeres function as sinks for retrotransposon insertion,^96^, ^97^, ^98^ we identified significant enrichment of retrotransposon insertions within centromeric alpha satellite HOR arrays.

Elevation of retrotransposon transcripts in Alzheimer's disease and progressive supranuclear palsy, a primary tauopathy, along with increased transposition in tau transgenic *Drosophila* ^12^, ^13^, ^14^, ^99^, ^100^, ^101^, ^102^ suggested that retrotransposons are derepressed and may mobilize in human disease. Our shallow (7X) long read sequencing reveals a trending increase in singleton/L1en‐mediated AluY insertions at Braak V/VI; deeper (30X) sequencing of neurons reveals a stronger, but still trending, increase in singleton/L1en‐mediated AluY insertions at Braak V/VI, suggesting that AluY elements may be more transpositionally active in Alzheimer's disease. New copies of retrotransposon DNA are generated via reverse transcription of retrotransposon RNA; the reverse transcriptase inhibitor lamivudine (3TC) reduces retrotransposition and neuronal death in laboratory models of Alzheimer's disease and related tauopathies.^13^, ^15^, ^102^, ^103^ In a Phase IIa clinical trial, 3TC was found to significantly reduce levels of glial fibrillary acidic protein (GFAP) in cerebrospinal fluid of individuals with early Alzheimer's disease, suggestive of reduced neuroinflammation.^104^ A retrospective analysis of public health records further suggests that reverse transcriptase inhibitors, including 3TC, decrease incidence of Alzheimer's disease.^105^ Given the low rate of L1en‐mediated singleton insertions detected in our analyses, most of which fall within centro/pericentromeric regions, we speculate that somatic retrotransposon *insertion* is not a primary factor driving neurodegeneration, and that blocking retrotransposon insertion is not the major mechanism underlying the protective effects of 3TC. As studies in *Drosophila* report that most new DNA copies generated via reverse transcription exist as extrachromosomal DNA,^106^ we speculate that retrotransposon‐derived extrachromosomal cytoplasmic DNA drives innate immune activation in Alzheimer's disease and related tauopathies, and that 3TC reduces neurotoxicity by limiting production of neuroinflammatory extrachromosomal DNA.

NAHR analyses revealed L1‐L1 recombination in alpha satellite monomeric repeats and Alu‐Alu recombination in centro/pericentromeric regions. While previous nanopore‐based analysis detected increased NAHR between retrotransposons in neurons in late‐stage Alzheimer's disease,^28^ the overall degree of NAHR did not differ between Alzheimer's disease cases vs. controls in our study. This discrepancy may result from neuron‐specific changes that are lost when DNA is extracted from total brain lysate. We nevertheless found that NAHR location differs at Braak III, with simple repeats/microsatellites particularly affected. We speculate that NAHR enrichment within repetitive regions at Braak III is due to loss of 5mC and/or other silencing mechanisms, thus increasing access to recombination machinery.

Array‐based quantification of DNA methylation in *post mortem* human Alzheimer's disease brain has identified increases and decreases in many different genes,^85^, ^92^, ^107^, ^108^ in line with our analysis of protein‐coding regions. In contrast, we found that methylation changes within repetitive regions were directionally consistent. 5mC methylation was globally decreased at Braak III, with centro/pericentromeric regions most affected; centro/pericentromeric 5mC was also reduced compared to chromosome arms at Braak V/VI. Studies utilizing late‐stage Alzheimer's disease brain report DNA hypomethylation based on 5mC immunoreactivity,^109^, ^110^ whereas array‐based approaches suggest DNA hypermethylation in Alzheimer's disease.^85^, ^111^, ^112^ We speculate that divergent findings result from the high degree of methylation within centro/pericentromeric regions of control brains, which is detected using immunoreactivity‐based approaches but is lost with arrays that do not include repetitive genomic regions. Previous array‐based platforms also cannot differentiate between 5mC and 5hmC, which is considered an activating DNA modification that occurs in opposition to 5Mc.^113^

Our findings add a new dimension to studies reporting loss of histone modifications and proteins associated with constitutive heterochromatin in tauopathy models and in neurons isolated from late‐stage human Alzheimer's disease brain.^114^, ^115^, ^116^ While we do not know the impact of centro/pericentromeric DNA demethylation in Alzheimer's disease, studies in cancer and Immunodeficiency, Centromeric Instability, and Facial Anomalies syndrome, a disorder caused by mutation of the centromere‐associated *DNA methyltransferase 3B* gene, suggest that DNA methylation regulates centromere function and stability by shaping local chromatin structure and suppressing recombination.^117^, ^118^, ^119^ As centro/pericentromeric demethylation is associated with aneuploidy and chromosomal rearrangements, we speculate that demethylation of these regions contributes to aneuploidy in Alzheimer's disease.^120^, ^121^, ^122^, ^123^ If centromeric demethylation induces production of centro/pericentromere‐derived RNA (cenRNA), such species could drive a senescence‐associated secretory phenotype.^124^

Aligned with studies in other tissues,^125^, ^126^ we identified rDNA arrays as hotspots for genomic variation. We detected numerous Alu‐Alu NAHR events within and between rDNA arrays, consistent with enrichment of DNA breakpoints near Alu sequences within rDNA of cancer cell lines.^127^ Presence of intrachromosomal rDNA NAHR events within 47S rDNA arrays suggest that rDNA duplications and deletions may stem from recombination of full‐length 47S rDNA copies and could underlie variations in rDNA copy number reported in other organisms.^128^, ^129^ Our finding that rDNA gene bodies are hypomethylated at early and late stages of Alzheimer's disease contrasts with a targeted bisulfite mapping‐based study reporting that rDNA promoters are hypermethylated in Alzheimer's disease.^130^ Differing findings may result from the inability of bisulfite sequencing to differentiate between 5mC and 5hmC. Future studies focused on rDNA NAHR, rDNA demethylation, and the potential for these mechanisms to produce circular rDNA^128^, ^131^ may reveal novel mechanisms that drive neurodegeneration, neuroinflammation, and senescence in Alzheimer's disease. Genomic changes in rDNA could also impact translation rate in settings of tauopathy^132^, ^133^, ^134^, ^135^ or translational fidelity.

While previous analysis of structural variants, often relying on DNA copy number and content, suggest that structural variation is elevated in Alzheimer's disease,^136^ these approaches can miss complex variants entirely. This results in a lack of information regarding structural variants in dark regions and/or retrotransposons that are in linkage disequilibrium with SNPs. We identified several insertions in a dark region composing a variable nucleotide repeat expansion highly correlated with a common high‐penetrant risk SNP (rs3764650) for Alzheimer's disease in *ABCA7*.^137^, ^138^ We also detect overlap between an Alzheimer's disease risk SNP (rs115550680) and a polymorphic AluYa8 element and identify several variants within other known risk genes. While these variants were not restricted to cases with tau pathology, our analyses reflect the tip of the iceberg regarding potential contributions of genetic variants within dark regions to disease. Future studies leveraging deeper long read sequencing and/or targeted sequencing methods in a larger set of individuals will enable population‐level variant analysis.^139^

Shallow sequencing combined with non‐cell‐type specific DNA sequencing presents several limitations. Low genomic coverage likely results in underestimation of genetic variants. For example, we detected a stronger trend of AluY retrotransposition events in individuals with Alzheimer's disease when DNA was sequenced at 30X compared to 7X coverage. Our overall estimated number of 5mC‐modified CpG sites (CHM13: 24.8 million, GRCh38: 32.28 million) is lower than the previously estimated number of CpG sites in the CHM13 (32.28 million) and GRCh38 human genome reference (29.17 million),^140^ likely reflecting relatively low coverage sequencing of our samples. Additionally, variation in cell type composition may obscure cell‐type‐specific methylation changes associated with disease; frontal cortex at Braak V/VI, for example, has less neurons and more glia than Braak 0 or III.^141^ Despite applying stringent criteria to remove false positives for singleton insertions and NAHR and manually inspecting each singleton insertion within alignment files, some observations may represent false positives. This could occur if sequenced reads at specific loci do not reflect the expected 50%/50% makeup of maternal vs. paternal genotype. False positives could result in inflated normalized sequencing values, resulting in estimates higher than those reported in the literature. Our analyses nevertheless provide new insight into genomic and epigenomic changes in Alzheimer's disease, as well as rationale for molecular and sequencing studies focused on genetic and epigenetic changes occurring in the dark genome.

## CONFLICT OF INTEREST STATEMENT

Bess Frost serves on the Scientific Advisory Board of Transposon Therapeutics. Other co‐authors have no competing interests. Author disclosures are available in the supporting information.

## CONSENT STATEMENT

Not applicable; *post mortem* human brain tissue was obtained from existing biorepositories.

## CODE AVAILABILITY

Example code used to generate data is deposited in the Zenodo repository (10.5281/zenodo.15831306).

## Supporting information



Supporting information

Supporting information

Supporting information

Supporting information

Supporting information

Supporting information

Supporting information

Supporting information

Supporting information

Supporting information

Supporting information

## Data Availability

All sequencing data is publicly available through the NCBI SRA database (PRJNA1083482).
